# Opioid-Induced Adrenal Insufficiency in a Patient Treated With Buprenorphine-Naloxone: A Case Report

**DOI:** 10.7759/cureus.72187

**Published:** 2024-10-23

**Authors:** Mohamed M Ahmed, Matthew League, Ramsey Amoudi, Clayton M Smith

**Affiliations:** 1 Internal Medicine, University of Louisville Hospital, Louisville, USA; 2 Internal Medicine, University of Louisville School of Medicine, Louisville, USA

**Keywords:** buprenorphine/naloxone, opioid epidemic, opioid-induced adrenal insufficiency, secondary adrenal insufficiency, suboxone side effects

## Abstract

With the rising clinical use of opioid agonist/antagonist medications for opioid use disorder (OUD), overlooked adverse events, such as adrenal insufficiency, are coming to the forefront. Opioid-induced adrenal insufficiency (OIAI) occurs when opioids dampen the hypothalamic-pituitary-adrenal (HPA) axis, leading to adrenal suppression. Opioid-induced adrenal insufficiency is associated with significant morbidity and can precipitate high-mortality adrenal crises. We present a case of a 33-year-old male patient, initially presenting with concern for gastrointestinal bleeding, in which Suboxone (buprenorphine/naloxone) was the likely culprit of a newly uncovered adrenal insufficiency.

## Introduction

Each year in the US, the opioid epidemic imposes a nearly $1.5 trillion economic burden and was responsible for a staggering 75% of the 107,543 overdose deaths occurring in 2023 [1,2]. One crucial way providers are addressing this is through the increasing use of opioid agonist/antagonist combination medications such as Suboxone (buprenorphine/naloxone) to treat opioid use disorder (OUD). With increasing use, we must be on the lookout for the emergence of previously thought rare adverse effects, as these may become more commonplace in clinical practice than previously observed.

One such important adverse effect is opioid-induced adrenal insufficiency (OIAI), a form of central adrenal insufficiency. Buprenorphine is a partial agonist at the Gi pathway-activated mu opioid receptors located in multiple areas throughout the body, including within the hypothalamus [3]. When hypothalamic mu opioid receptors are activated, we see the reduced activity of the hypothalamic-pituitary-adrenal (HPA) axis, thus low administration of cosyntropin (ACTH) and cortisol levels. Buprenorphine dampening the adrenal response has been well illustrated in animal subjects as well as measured in humans via reduction of salivary cortisol directly relating to human stress response [4,5]. 

Opioid-induced adrenal insufficiency is associated with significant morbidity and typically presents with nonspecific symptoms such as anorexia, nausea, vomiting, abdominal pain, weakness, fatigue, lethargy, and fever [6,7]. The inability to produce sufficient quantities of cortisol can precipitate life-threatening adrenal crises, which can significantly increase patient mortality, particularly in those with concomitant cardiac, pulmonary, or infectious diseases [8]. The diagnosis of OIAI is confirmed with an ACTH stimulation test. Resolution of OIAI can be achieved through cessation or reduction of opiate medications and symptomatic treatment via glucocorticoid replacement. With the growing demand for opioid cessation treatments and a gap in clinical awareness, this report aims to draw attention to this rare but serious side effect.

## Case presentation

A 33-year-old male with a medical history of treated hepatitis C, opioid use disorder (currently managed with Suboxone), iron deficiency anemia (IDA), leukopenia, neutropenia, history of positive lupus anticoagulant, foreign body ingestion, and self-mutilation presented to the emergency department with complaints of diffuse abdominal pain, hematemesis, and melena. Initial laboratory investigations revealed a critically low hemoglobin level of 3.7 g/dL. He was admitted to the ICU, where he received four units of packed red blood cells and was hemodynamically stabilized. A gastroenterology consultation was obtained, and both an upper endoscopy and colonoscopy conducted were unrevealing for a source of bleeding. Repeat iron panel significant for IDA, and the patient received four days of ferric gluconate 250 mg daily. 

The patient's substance use history was notable for extensive opioid use. According to the patient, he had been using Percocet (30 mg tablets) up to five to six times daily for substance abuse before transitioning to Suboxone treatment. He was initiated on buprenorphine-naloxone (Suboxone) 8-2 mg sublingual tablets twice daily in March 2023 and has since denied any further use of Percocet.

On the third day of hospitalization, the patient developed recurrent hypotension that was refractory to intravenous fluid resuscitation. The medical team conducted a comprehensive evaluation, ruling out common etiologies, including septic shock and hypothyroidism. Clinically, the patient remained afebrile, without tachycardia or tachypnea, and his chest X-ray was negative for pneumothorax or signs of pneumonia. A bedside echocardiogram demonstrated euvolemia and preserved cardiac contractility, consistent with a recent formal echocardiogram from December 2023, which showed a normal left ventricular ejection fraction (LVEF) of 65% and a patent foramen ovale.

**Figure 1:**
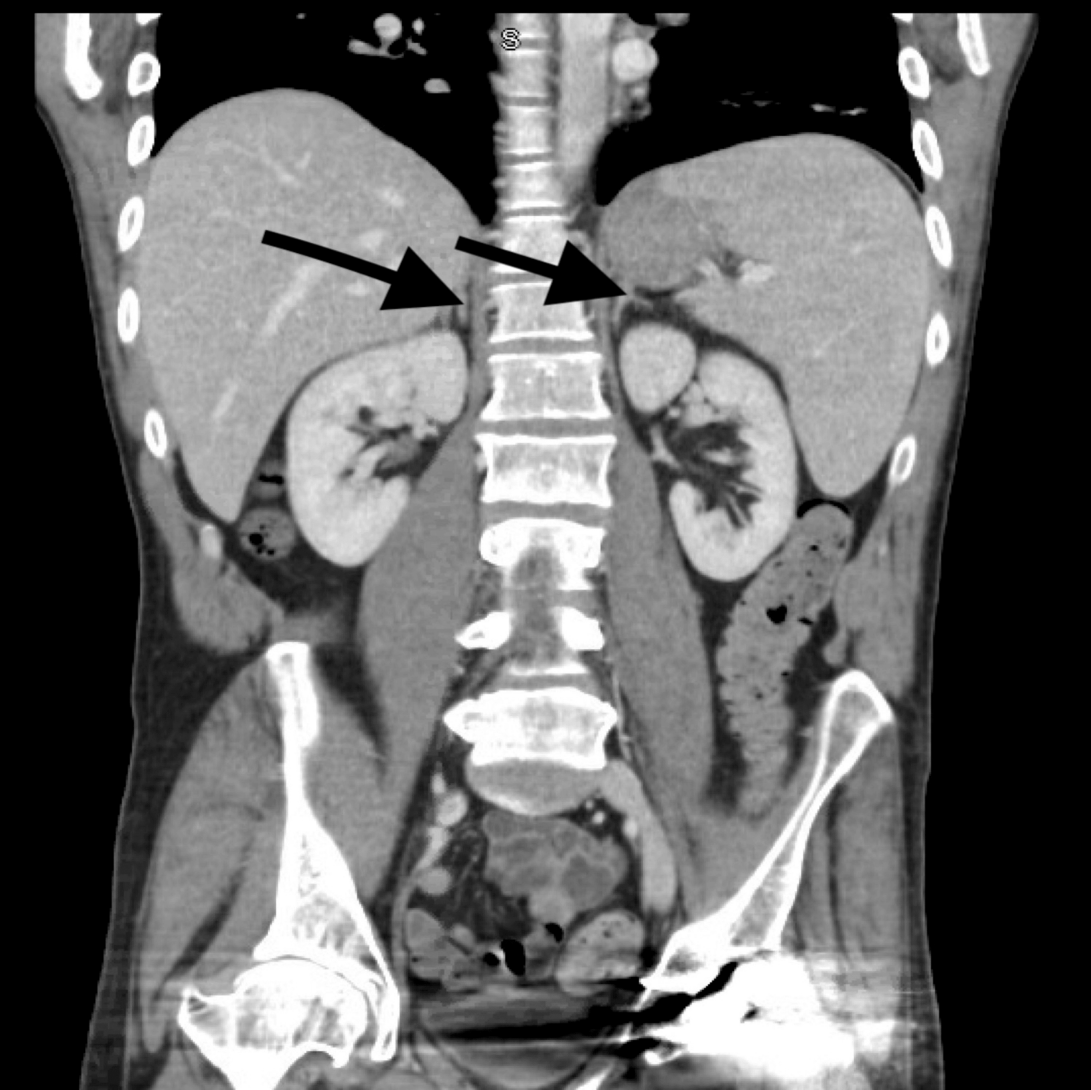
Coronal view of the CT scan of the abdomen and pelvis; arrows indicate normal-sized left and right adrenal glands

Given the patient’s persistent hypotension and inconclusive initial workup, adrenal insufficiency was suspected. A morning serum cortisol level of 1.4 mcg/dL was obtained, and an ACTH stimulation test demonstrated a suboptimal response, with cortisol levels rising from 2.8 mcg/dL to only 17 mcg/dL following cosyntropin administration, leading to a presumptive diagnosis of secondary adrenal insufficiency (Table 1). A CT scan of the head without contrast revealed a pituitary gland of normal size and appearance, with no evidence of enlargement or masses. Additionally, an abdominal CT scan showed unremarkable adrenal glands (Figure 1). Given the patient’s history of lupus anticoagulant disorder, IDA, and leukopenia, a hematology/oncology consultation was obtained. The immunologic workup yielded negative results, and the team concluded that the leukopenia and anemia were transient, as both white blood cell counts and hemoglobin levels improved during hospitalization. An anemia workup, conducted two days post-transfusion, revealed microcytic anemia with normal ferritin levels likely normalized following the blood transfusion. Laboratory values are provided in Table 1.

**Table 1:** Pertinent laboratory values and imaging findings WBC: white blood cell; Hgb: hemoglobin; Plt: platelets; Eos: eosinophils; MCV: mean corpuscular volume; TIBC: total iron binding capacity; PBS: peripheral blood smear; IDA: iron deficiency anemia; TSH: thyroid-stimulating hormone; H: high; L: low; AM: morning; Hep: hepatitis; Ab: antibodies; PTT-LA: partial thromboplastin time – lupus anticoagulant; dRVVT: diluted Russell viper venom time

Test	Hospital day	Result	Reference range
Urine opiates	2	Positive	Negative
Urine oxycodone	2	Negative	Negative
Urine cocaine	2	Negative	Negative
Urine methadone	2	Negative	Negative
Urine fentanyl	2	Negative	Negative
Sodium (mmol/L)	3	135 L	135–145
Potassium (mmol/L)	3	4.1	3.5–5.0
WBC (thou/mcL)	3	3.1 L	4–11
Hgb (g/dL)	3	9.3 L	13.2-16.6
MCV (fL)	3	76.8	80-100
Plt (x10^3/uL)	3	385	150-450
Eos count (uL)	3	0.1	0.0-0.6
Serum Iron (ug/dL)	3	188	29-165
Ferritin (ng/dL)	3	116	7-350
TIBC (ug/dL)	3	395	252-461
Transferrin (mg/dL)	3	282	180-329
Haptoglobin (mg/dL)	3	85	35-195
Retic absolute (million/mm^3)	3	0.13	0.02-0.1
PBS	3	Notable for burr cells and some hyperchromasia commonly seen in patients with liver dysfunction and IDA, respectively. No other acute findings were noted.	NA
TSH µU/mL	1	0.78	0.5-5
Lactic acid mmol/L	2	0.6	0.5-1.9
Cortisol (mcg/dL) *AM draw	3	1.4 L	5-25
Cortisol (mcg/dL) *AM draw	4	2.8 L	5-25
Cortisol (mcg/dL) *60min post Cosyntropin draw	4	17 L	≥18
Hep A IgM AB	2	Nonreactive	Nonreactive
Hep B surface AG	2	Nonreactive	Nonreactive
Hep B Core AB IgM	2	Nonreactive	Nonreactive
Hep C AB	2	Reactive (A)	Nonreactive
Hep C RNA	2	Not Detected	Non Detected
HIV 1 & 2	2	Non-reactive	Non-reactive
Beta-2 glycoprotein 1, IgA	2	<2	0 - 20 U/mL
Beta-2 glycoprotein 1, IgG	2	<1.4	0 - 20 U/mL
Beta-2 glycoprotein 1, IgM	2	4.8	0 - 20 U/mL
Complement C3	2	107	90 - 180 mg/dL
Complement C4	2	37	10 - 40 mg/dL
ACA IgG	2	<1.6	0 - 20 GPL
ACA IgA	2	<2	0 - 20 GPL
ACA IgM	2	2	0 - 20 GPL
Anti Ps/PT IgG	2	<9	<40 U/mL
Anti Ps/PT IgM	2	23	<40 U/mL
B2 glycoprotein 1 (IgG) Ab	2	<2	0 - 20 U/mL
B2 glycoprotein 1 (IgA) Ab	2	<2	0 - 20 U/mL
B2 glycoprotein 1 (IgM) Ab	2	4.1	0 - 20 MPL
Cardiolipin Ab (IgA)	2	<2	0 - 20 MPL
Cardiolipin Ab (IgG)	2	<2	0 - 20 MPL
Cardiolipin Ab (IgM)	2	<2	0 - 20 MPL
Lupus anticoagulant	2	Detected in 6/2024; negative on 7/28/2024	Negative
PTT-LA screen	2	33	25 - 35 seconds
dRVVT screen	2	36, 35	31 - 44 seconds
CT of the head without IV contrast	0	No acute intracranial abnormalities were noted. The pituitary gland was normal in size and appearance, with no enlargement or masses.	NA
CT of the abdomen and pelvis with IV contrast	0	No hematoma or evidence of active bleeding. No pneumoperitoneum. No other acute findings in the abdomen and pelvis. No mass in the adrenal glands. Incidental musculoskeletal findings include moderately severe right hip osteoarthritis, bilateral spondylolysis defects at L5, and possible mild old compression fractures from T8 through T11.	NA

Consequently, Suboxone was discontinued, and the patient was initiated on hydrocortisone therapy at 10 mg twice daily. Following the introduction of hydrocortisone, the patient’s blood pressure normalized to 115/67 mmHg (Table 2). He demonstrated clinical improvement but chose to leave against medical advice. The patient was advised to follow up with an outpatient endocrinologist. Unfortunately, we were unable to reassess adrenal recovery as the patient was lost to follow-up. 

**Table 2:** Vitals before and after initiating hydrocortisone

Vitals signs	Before starting hydrocortisone	After starting hydrocortisone	Reference range
Temperature (°C)	Afebrile	Afebrile	36.1-37.2°C
Blood pressure (BP)	90/48 mmHg	115/67 mmHg	90/60-120/80 mmHg
Heart rate (HR)	87 bpm	77 bpm	60-100 bpm
Respiratory rate (RR)	18 breaths/min	18 breaths/min	12-20 breaths/min
Oxygen saturation (SpO2)	95% (room air)	97% (room air)	≥95% on room air

## Discussion

Opioid-induced adrenal insufficiency is a recognized complication of chronic opioid therapy, with a recent meta-analysis estimating a prevalence of approximately 15% among long-term opioid users [9]. Morphine, fentanyl, and oxycodone are the most commonly implicated opioids. Here, we present a unique case of OIAI induced by buprenorphine/naloxone, a partial agonist at opioid receptors and the primary component of Suboxone. Although cases of buprenorphine-induced OIAI have been reported, such instances remain exceedingly rare, and comprehensive data on Suboxone-induced OIAI are limited in the current literature.

Chronic opioid use is known to suppress the HPA axis via G-protein-coupled mu-opioid receptors, leading to decreased adrenal stimulation and, ultimately, adrenal insufficiency. This suppression poses a significant risk of adrenal crisis, especially in patients with existing cardiac, pulmonary, or infectious diseases.

The OUD epidemic has emerged as a significant public health crisis in the United States, causing substantial financial strain and numerous fatalities. Evidence-based treatments, including methadone and Suboxone, have proven effective for managing OUD. The 2008 Clinical Trials Network (CTN) Study demonstrated Suboxone's safety and efficacy, showing a significant reduction in illicit opioid use and improved retention in treatment [10].

As a result, buprenorphine prescriptions have risen markedly. From 2019 to 2022, national buprenorphine prescription rates surged to 4.8 per 100 people, with over 16 million prescriptions in 2022 [11]. Initially, only physicians with an X-waiver could prescribe Suboxone. However, as the demand for OUD treatment grew, the U.S. Department of Health and Human Services relaxed X-waiver requirements in April 2021 and was eliminated on January 12, 2023. Consequently, a recent study by University of Michigan researchers published in the New England Journal of Medicine revealed a significant rise in buprenorphine prescribers following a federal policy change. By December 2023, over 53,600 clinicians were prescribing buprenorphine, marking an increase of 11,500 compared to December 2022 [12]. 

Suboxone was traditionally initiated in outpatient settings. However, recent studies indicate that starting buprenorphine treatment during hospitalization is associated with reduced overdose risk and improved post-discharge treatment engagement [13, 14]. The American Society of Addiction Medicine (ASAM) and the Substance Abuse and Mental Health Services Administration (SAMHSA) now recommend initiating medication-assisted treatment (MAT), including Suboxone, even in inpatient settings to improve continuity of care. This has led to a significant increase in the number of inpatient physicians prescribing Suboxone before patient discharge.

Despite Suboxone's benefits, OIAI induced by Suboxone remains a rare and often under-recognized condition. With the increasing number of Suboxone prescriptions, the risk of missed OIAI cases could rise, underscoring the urgency for increased awareness. A recent study highlighted that endocrinologists are more knowledgeable about opioid-induced endocrinopathies, with 69% recognizing these conditions, compared to just 24% of non-endocrine providers. Furthermore, 38% of endocrinologists could correctly identify OIAI symptoms, whereas only 9% of non-endocrine providers could do so. Additionally, 25% of non-endocrine providers reported discomfort in managing glucocorticoid replacement therapy for OIAI patients [15].

These findings underscore the urgent need for increased education and awareness of OIAI among healthcare providers, particularly non-endocrinologists. The development of comprehensive guidelines, educational resources, and training materials is essential to improving clinicians' ability to recognize, diagnose, and manage OIAI effectively. This approach is critical to ensuring timely identification and treatment, especially as Suboxone use continues to expand, and will provide healthcare providers with the necessary support and tools.

## Conclusions

This case highlights a rare but clinically significant instance of buprenorphine/naloxone-induced adrenal insufficiency (OIAI) in a patient treated with Suboxone for OUD. With the increasing use of buprenorphine in managing OUD, healthcare providers must remain vigilant for potential adverse effects like OIAI. Early identification and prompt treatment with glucocorticoid replacement can be life-saving, as evidenced by the resolution of hypotension and clinical improvement in our patient. Given the nonspecific nature of OIAI symptoms, heightened awareness and education among clinicians, especially those managing OUD, are crucial. Physicians treating patients on any opioid-containing medications, including partial opioid agonists, should maintain a low threshold for testing for OIAI in individuals presenting with classic symptoms of adrenal insufficiency, such as nocturnal hypotension, dizziness, anorexia, or fatigue. This case serves as a critical reminder that OIAI secondary to Suboxone, though rare, should be considered in patients experiencing unexplained symptoms while on chronic opioid therapy.
